# Gathering Novel Circulating Exosomal microRNA in Osteosarcoma Cell Lines and Possible Implications for the Disease

**DOI:** 10.3390/cancers11121924

**Published:** 2019-12-03

**Authors:** Nicola Cuscino, Lavinia Raimondi, Angela De Luca, Claudia Carcione, Giovanna Russelli, Laura Conti, Jacopo Baldi, Pier Giulio Conaldi, Gianluca Giavaresi, Alessia Gallo

**Affiliations:** 1 IRCCS ISMETT (Istituto Mediterraneo per i Trapianti e Terapie ad alta specializzazione), Research Department, 90127 Palermo, Italy; ncuscino@ismett.edu (N.C.); grusselli@ismett.edu (G.R.); pgconaldi@ismett.edu (P.G.C.); 2 IRCCS ISTITUTO ORTOPEDICO RIZZOLI, 40136 Bologna, Italy; lavinia.raimondi@ior.it (L.R.); angela.deluca@ior.it (A.D.L.); gianluca.giavaresi@ior.it (G.G.); 3 Fondazione Ri.MED, 90133 Palermo, Italy; ccarcione@fondazionerimed.com; 4 Body Fluids Biobank, Clinical Pathology, IRCCS Regina Elena Cancer National Institute, 00100 Rome, Italy; laura.conti@ifo.gov.it; 5 Dept. of Orthopaedic Oncology, IRCCS Regina Elena National Cancer Institute, 00100 Rome, Italy; jacopo.baldi@ifo.gov.it

**Keywords:** microRNAs, exosomes, liquid biopsy

## Abstract

One of the goals of personalized medicine is to understand and treat diseases with greater precision through the molecular profile of the patient. This profiling is becoming a powerful tool for the discovery of novel biomarkers that can guide physicians in assessing, in advance, the disease stage, and monitoring disease progression. Circulating miRNAs and exosomal miRNAs, a group of small non-coding RNAs, are considered the gold standard diagnostic biomarkers for human diseases. We have previously demonstrated that osteosarcoma-derived exosomes are able to influence crucial mechanisms inside tumor niches, inducing osteoclast differentiation, and sustaining bone resorption activity. Here we discovered, through Next-Generation Sequencing (NGS), eight novel microRNAs in three different osteosarcoma cell lines, and assessed the selective packaging into the exosomes released. We then investigated, as proof-of-principle, the presence of the novel microRNAs in osteosarcoma patient samples, and found that 5 of the 8 novel microRNAs were more present in circulating exosomes of osteosarcoma patients compared with the controls. These results raise a question: Could the 8 novel microRNAs play a role for osteosarcoma pathogenesis? Although still premature, the results are encouraging, and further studies with a validation in a larger cohort are needed.

## 1. Introduction

Next-generation sequencing (NGS) and liquid biopsies, also through circulating microRNAs (miRNAs), are both tools that may provide therapeutic strategies to oncologists, which contribute to the development of precision medicine. Several research groups have already investigated circulating miRNAs for their diagnostic and prognostic potential, suggesting them as useful cancer biomarkers [1,2,3,4]. In osteosarcoma (OS), some clinical studies have investigated the prognostic and diagnostic potential of microRNAs [5,6]. Moreover, the identification of novel circulating miRNAs, released through exosomes into the blood from malignant cells, can provide novel biomarkers and therapeutic targets for cancer patients [6]. OS is the most common type of primary bone tumor of the skeleton, occurring mainly in children and adolescents, in the metaphyseal region of the long bones, typically in the distal femur, proximal tibia, and humerus [7].

Multidisciplinary treatment, combined with surgery for localized tumors, have led to a five-year survival rate of 60–70% in non-metastatic patients. However, approximately 20% of patients diagnosed with metastatic OS at presentation, primarily in the lungs, are instead characterized by a worse clinical outcome [8]. Among the novel approaches that do not require surgical biopsy, liquid biopsy is useful in improving the prognosis, and monitoring disease course and survival rates of OS patients, offering information on micro-metastasis at diagnosis and minimal residual disease, the latter only partially detectable by conventional diagnostic methods [9].

In our previous study, we highlighted, by RNA sequencing methods, a packaging of specific miRNAs within OS cell-derived exosomes. In detail, we first demonstrated the role of OS cell-derived exosomes, inside the tumor microenvironment, in bone metabolism and tumor angiogenesis; we then focused our attention on some of these specific exosomal miRNAs, and demonstrated their involvement in osteoclast differentiation, bone resorption activity, and angiogenesis [10].

In the present study, in taking advantage of the NGS approach, we discovered and analyzed the expression of eight novel miRNAs in OS cell lines and OS cell-derived exosomes. We also analyzed their expression in a panel of human tissues and in a small group of OS patients. Notably, we found selective packaging of some of these novel miRNAs into the exosomes released by OS cells and into the circulating exosomes from plasma of OS patients. Although, the functional role of exosome-encapsuled miRNAs must be investigated in depth, the results obtained lead us to hypothesize a role for these novel miRNAs as having a circulating biomarker potential in OS, and for enabling personalized treatments in precision medicine.

## 2. Results

### 2.1. RNA Profiles of Osteosarcoma Cell Lines and Osteosarcoma Cell-Derived Exosomes

In order to discover novel microRNA specific for OS, we start with three OS cell lines: SAOS-2, MG-63, U-2 OS, and the exosomes released by those cell lines. Exosomes were isolated and characterized as previously described [10]. After assessing the RNA quality, we performed small RNA sequencing on the MG-63 cell line and MG-63 exosomes; the U-2 OS cell line and U-2 OS exosomes; and the SAOS-2 cell line and SAOS-2 exosomes. Due to the NGS approach, we were able to determine, not only the differences in the expression of known miRNAs in the parent cells and the exosomes, but also novel sequences expressed among the different cell lines in relation to their exosome miRNA cargo. Novel miRNA profiles were analyzed in three OS cell lines: MG-63, U-2 OS, and SAOS-2, and their exosomes through miRDeep2. 3116, 5916, and 3381, novel putative miRNA sequences from MG-63, SAOS-2, and U-2 OS, respectively, remained after filtering for known contaminant and highly common sequences, such as rRNA and tRNA, for all known miRNA sequences from miRBase v22 and for exon (https://www.ncbi.nlm.nih.gov/sra/PRJNA575520). We further filtered the miRDeep2 results by score. MiRDeep2 scores ranged from -10 to 10, with a higher number corresponding to increased likelihood that the miRNA is genuine. A cut-off of 0 was used to be included in this study. We chose to validate two miRNAs for MG-63, three for U-2 OS, and three for SAOS-2, with the highest raw counts for each cell line (Table 1). In Supplementary Table S1 and Supplementary Material 2, we report the sequences, the genomic locations and predicted structures of the 8 pre-miRNA candidates.

### 2.2. Validation of the Candidates’ Novel microRNAs

**Figure 1:**
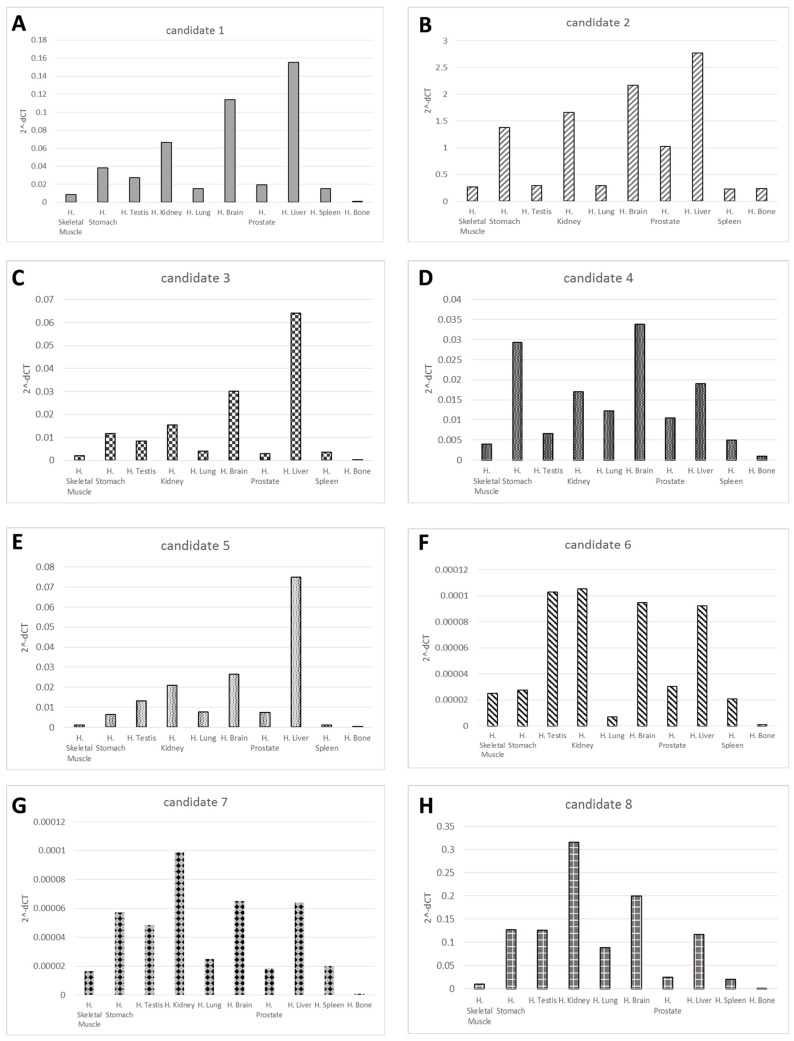
Novel candidate miRNA validation. The graphs (**A**–**H**) show quantitative RT-PCR results of the 8 novel candidate miRNAs expression, respectively, in 10 different human tissue types. Results are displayed as mean levels to the average expression reported as 2^^-dCT^, and are normalized to U6.

Once we obtained the 8 candidate microRNA sequences (Table 1) with a high score through miRDeep2, we used custom Taqman assays to first validate on the SAOS-2, MG-63, and U-2 OS cell lines and their exosomes (Supplementary Figure S1). We then measured the expression of the candidate microRNAs across a panel of 10 different human tissue RNA (skeletal muscle, stomach, testis, kidney, lung, brain, prostate, liver, spleen, and bone) to assess the presence in different body parts and eventually the tissue specificity. Custom Taqman assays gave reproducible and consistent results, and were able to amplify the target novel miRNAs in most of the tissue types tested. The liver, kidney, and brain RNAs showed the highest candidate microRNAs expression, while skeletal muscle, lung, spleen and bone showed the lowest candidate microRNAs expression (Figure 1).

### 2.3. Candidate Novel microRNA Expression Circulating in Osteosarcoma Samples

Once validated in different human tissues, we wanted to investigate the presence of the candidate microRNAs discovered in OS cell lines, and eventually the differential in samples of OS patients. As proof-of-principle, to test the prognostic potential of the novel candidate microRNAs in liquid biopsies, we analyzed plasma from 5 OS patients whose clinical features are reported in Table 2, and 3 controls by digital PCR. 

**Figure 2:**
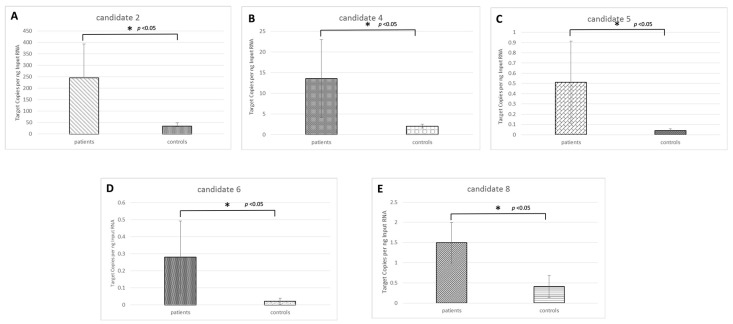
Novel candidate miRNAs expression in osteosarcoma patient samples. The graphs (**A**–**E**) show quantitative RT-PCR results of the novel candidate miRNAs 2/4/5/6/8 respectively that resulted highly expressed circulating in osteosarcoma sample compared with the controls. Results are displayed as mean levels to the average expression calculated as Target Copies per ng Imput RNA, and are normalized to U6. One star (*) indicates *p* < 0.05, two-tailed Student’s test.

We choose this approach because of such advantages as the partitioning of the PCR reaction into thousands of individual reactions; the end-point measurement enables nucleic acid quantitation independent of the reaction efficiency. In this set of samples, 5 novel candidate microRNAs (candidates 2, 4, 5, 6, and 8) out of eight showed a significant differential expression in OS samples compared with the controls (Figure 2). One novel candidate microRNA (candidate 7) did not amplify in any sample; one novel candidate microRNA (candidate 3) showed no difference between the two groups, and novel candidate 1 showed the highest expression in the control group compared with the OS group.

We further determined the biologic pathways affected by novel candidate miRNAs using TargetScan web platform able to predict biological targets of microRNAs by searching for the presence of sites that match the seed region of each miRNA. We reported in Table 3 the KEGG biological processes significantly enriched (pathways with *p* < 0.05). All clearly involved with carcinogenesis for each candidate microRNAs determined by Enrichr web platform.

## 3. Discussion

In the management of OS, a proper diagnosis and staging of the disease is a major prerequisite for effective surgical and pharmacological treatments. Conventional diagnostic approaches, such as tissue biopsy and imaging, remain the most common diagnostic tests. Nevertheless, tissue biopsy may sometimes be difficult to obtain and not be easily repeatable. In addition, information concerning micro-metastasis or minimal residual disease can sometimes be lost with conventional diagnostic methods [11]. Liquid biopsy may be highly advantageous, given the difficulty of recovering the bone sample for disease staging. In fact, it is a non-invasive and also time-saving approach; moreover, it may offer more precise and accurate information on early detection, therapeutic decisions, and response to therapy in OS [12,13].

In this paper we identified eight novel microRNAs in OS cell lines (MG-63, SAOS-2, and U-2 OS) and their released exosomes. The eight sequences remained after filtering for known contaminant from 3116, 5916, and 3381, novel putative miRNA sequences, obtained by MG-63, SAOS-2, and U-2 OS, respectively. We chose to validate two miRNAs for MG-63, three for U-2 OS, and three for SAOS-2, with the highest raw counts for each cell line. To assess the tissue specificity, we analyzed, by custom TaqMan assays, the expression of the eight candidate microRNAs across a panel of 10 different human tissue RNAs (skeletal muscle, stomach, testis, kidney, lung, brain, prostate, liver, spleen and bone). The candidate microRNAs were found highly expressed in the liver, kidney, and brain, while skeleton muscle, lung, spleen and bone showed the lowest candidate microRNAs expression. 

To fill the current gaps and needs advanced by clinical oncologists, we decided to investigate the presence of the novel candidate microRNAs circulating in OS patients, and compare them with controls. Five of eight novel candidate microRNAs were found highly and significantly expressed in OS samples compared with the controls. This fact, together with the low expression of these microRNAs in the healthy bone tissue, make the novel candidates potential disease biomarkers.

Through TargetScan web platform, we identified the putative genes regulated by these five candidate microRNAs. We further determined, through the web platform Enrichr, the biologic pathways predicted and potentially affected. The KEGG biological processes significantly enriched (pathways with *p* < 0.05), reported in Table 3, resulted clearly involved with carcinogenesis for each candidate; Among these, candidate microRNA 5 is predicted to bind and strongly regulate VEGFA which expression is crucial for OS growth and metastasis [14]. Another example is candidate microRNA 2, predicted to bind SMAD2 and SMAD4, genes already related with Osteosarcoma [15], and resulting potentially interesting for further in vitro studies.

Overall, these results allowed us to hypothesize that these 8 novel candidate miRNAs could potentially represent biomarkers for OS. As a result, we plan to carry out future clinical studies on large cohorts of OS patients in order to better analyze and compare the expression of novel miRNAs in patient subgroups, distinguishing metastatic patients from non-metastatic ones. We suppose that such information could correlate the expression of miRNAs with the disease progression. At the same time, functional studies, by in vitro assays, may shed light on the molecular mechanisms beyond the possible pathologic role of these microRNAs.

## 4. Materials and Methods

### 4.1. Ethics Approval

The Bioethics Committee of the IFO_AOO - AOO - Istituti Fisioterapici Ospitalieri on February 16, 2017 (resolution number899/17) or osteosarcoma patients approved this study. 

The aim of the study, study stages, and sample collection procedures were explained to all subjects. All subjects gave their written informed consent to participate in the study, including for blood sample collection and use of clinical data for research.

### 4.2. Cell Lines and Reagents

SAOS-2, MG-63, and U-2 OS cell lines were purchased from ECACC (Sigma-Aldrich, Milano, Italy), and grown in DMEM high glucose (Thermo Fisher Scientific, Cambridge, MA, USA) supplemented with 10% fetal bovine serum (FBS, Lonza Group, Basel, Switzerland).

### 4.3. Exosome Purification

Exosomes released by OS cells (SAOS-2, MG-63, and U-2 OS) during a 48-hour culture period were isolated from conditioned culture medium supplemented with 10% FBS, previously ultra-centrifuged by differential centrifugations as previously described. Exosome protein content was determined by the Bradford assay [16]. Exosomes from plasma of OS patients were isolated with Total Exosome Isolation Kit (Thermo Fisher Scientific, Cambridge, MA, USA), according to the manufacturer’s instructions.

### 4.4. Small RNA Library Construction and Sequencing

To test the quality and assess the quantity of total RNA extracted, we used the RNA ScreenTape assay on a 2200 TapeStation system (Agilent Technologies, Santa Clara, CA, USA). For small RNA-Seq, 1 μg of total RNA per sample was used for library preparation using TruSeq Small RNA Sample Prep Kits (Illumina, San Diego, CA, USA). Size-distribution was measured with the DNA ScreenTape assay on a 2200 TapeStation system (Agilent Technologies, Santa Clara, CA, USA). A total library pool of 4 nM was sequenced using a MiSeq Reagent Kit v3 150 cycle on a MiSeq System (Illumina, San Diego, CA, USA).

### 4.5. Small RNA-Seq in Silico Analysis

We used the Trimmomatic software [17], v. 0.3633 to remove adaptors, low-quality bases, and reads with less than 16 nucleotides. The parameter “ILLUMINACLIP TruSeq3-SE.fa:2:30:10” was used to remove read adaptors according to Illumina-specific sequences. A sliding window cut was applied to remove bases with average quality below 22 using the parameter “SLIDINGWINDOW:3:22,” and reads with less than 16 nucleotides were removed using “MINLEN:16.”

Alignment of miRNA sequencing reads to the human reference genome build hg19 was performed using Bowtie v. 1.2.2 [18]. More stringent read length filtering was carried out by miRDeep2 [19] before the identification of novel miRNAs, discarding reads with length less than 18 nucleotides and greater than 23 nucleotides. We used the miRDeep2 v.2.0.1.1 software (Berlin Institute for Medical Systems Biology at Max-Delbrück-Center for Molecular Medicine, Berlin-Buch, Germany) default parameters, using the Fasta format sequences of all mature and hairpin miRNA sequences obtained from miRBASE v.22 database.

### 4.6. Real-time PCR Validation and Digital PCR of Novel microRNAs

For miRNA validation, total RNA from 10 human tissues (H. Skeletal Muscle, H. Stomach, H. Testis, H. Kidney, H. Lung, H. Brain, H. Prostate, H. Liver, H. spleen from Gentaur S.r.l. Italy and H. Bone from OriGene Technologies GmbH, MD, USA) were reverse transcripted with TaqMan MicroRNA Reverse Transcription Kit, according to the manufacturer’s instructions (Thermo Fisher Scientific, Cambridge, MA, USA). Custom TaqMan microRNA Assay are listed in Supplementary Table S2). Novel candidate miRNA expression results are displayed as 2^^−dCT^, and are normalized to U6 (Thermo Fisher Scientific, Cambridge, MA, USA). Twenty ng of RNA extracted from plasma of OS patients and controls were reverse transcripted with TaqMan MicroRNA Reverse Transcription, according to the manufacturer’s instructions (Thermo Fisher Scientific, Cambridge, MA, USA). The Droplet Digital™ PCR (ddPCR) reactions were prepared in 20 μL total volumes, according to the manufacturer’s instructions (Bio-Rad Laboratories, Irvine, CA, USA). The thermal cycling conditions of the droplets generated were as follows: 95 °C for 10 min (1 cycle); then 40 cycles of 94 °C for 30 s, and 60 °C for 1 min, 98 °C for 10 min, and then held at 4 °C. After thermal cycling, droplets were analyzed for positive and negative signals using the QX200 droplet reader (Bio-Rad Laboratories, Irvine, CA, USA). Data analysis was done when the number of droplets produced was more than 20,000. For data analysis, QuantaSoft Version 1.7.4 software (Bio-Rad Laboratories, Irvine, CA, USA) was used to statistically analyze the obtained data. Novel candidate miRNA expression was normalized on U6 (Thermo Fisher Scientific, Cambridge, MA, USA) and the results are displayed as Target Copies per ng Imput RNA [20].

### 4.7. miRNA Pathway Analysis

To identify potential target genes and pathways of the differentially expressed novel candidate miRNAs found, we conducted in silico analysis using TargetScan web platform [21], in order to identify the putative genes and Enrichr web platform [22] to determine the putative pathways.

## 5. Conclusions

We have identified eight novel microRNAs, through NGS, in three osteosarcoma cell lines and demonstrated the selective packaging into their released exosomes. Below, the expression of the novel miRNAs was confirmed in circulating exosomes from plasma of osteosarcoma patients. The exact role of the novel miRNAs will be assessed through future clinical and molecular studies. The data obtained suggest these novel miRNAs as having a circulating biomarker potential in osteosarcoma cancer, assuming a role in personalized medicine.

## Figures and Tables

**Table 1:** Sequence and location information for the eight candidate miRNAs.

Novel ID	Location (hg19)	Strand	Mature Sequence
Candidate 1	chr9:136204572..136204654	−	CCCCACACUGCUAAAUUUGAC
Candidate 2	chr3:14436198..14436257	+	GGAAUAACGGGUGCUGUAGGCU
Candidate 3	chr9:89037929..89037970	−	CCCCUCACUGCUAAAUUUGAC
Candidate 4	chrX:102411053..102411132	−	CCAUCUGUGGGAUUAUGACUGA
Candidate 5	chr16:33962833..33962878	+	UGCGCAGUGGCAGUAUCGUAGCC
Candidate 6	chr8:56821958..56822010	−	UAUGUGCCUAGUGGCUGCUGUCU
Candidate 7	chr13:27259470..27259536	+	UCUGGGCAACAAGGUGAGACC
Candidate 8	chr9:89037927..89037972	−	AUGGAUUUUUGGAAAUAGGAGA

**Table 2:** Clinical characteristics of Osteosarcoma patients.

Patient ID	Gender	Age	Site of Origin	Histologic Subtype	metastasis	Treatments
Patient 1	M	14	sx prox Tibia	High Grade Surface OS	No	Chir
Patient 2	M	17	dx distal Femur	OS G3	No	CHT (CDDP/ADM MTX HD × 2)
Patient 3	M	18	dx prox Tibia	OS condrobastic G3	No	CHT (CDDP/ADM MTX HD × 2)
Patient 4	M	19	sx Tibia	OS fibroblastic	Lung	CHT (CDDP/ADM MTX HD × 2)
Patient 5	M	16	dx Pelvis	OS G3	Lung	CHT (CDDP/ADM MTX HD × 2)

**Table 3:** Biologic pathways enriched by differentially expressed novel candidate miRNAs.

**Candidate 2**	**KEGG pathway**	***p*-value**	**Genes**
	FoxO signaling pathway	6.37 × 10^−5^	*SMAD2;SMAD4;CCND2;MAPK1;PIK3R1;IGF1*
	TGF-beta signaling pathway	1.208 × 10^−3^	*SMAD2;SMAD4;MAPK1;ACVR1B;BMPR1B;SKP1*
	Hippo signaling pathway	1.29 × 10^−3^	*FZD1;SMAD2;PRKCI;SMAD4;CCND2;NF2*
	Proteoglycans in cancer	1.41 × 10^−3^	*FZD1;SMAD2;PDCD4;MAPK1;PIK3R1;IGF1*
	Hepatocellular carcinoma	1.75 × 10^−3^	*FZD1;SMAD2;SMAD4;CDK6;MAPK1;PIK3R1*
	Glioma	3.09 × 10^−3^	*CDK6;MAPK1;PIK3R1;IGF1;CALM1*
	Pancreatic cancer	3.09 × 10^−3^	*SMAD2;SMAD4;CDK6;MAPK1;PIK3R1*
	Breast cancer	3.35 × 10^−3^	*FZD1;JAG2;CDK6;MAPK1;PIK3R1;IGF1;ESR1*
**Candidate 4**	**KEGG pathway**	***p*-value**	**Genes**
	Cell adhesion molecules	3.61 × 10^−4^	*NLGN3;CNTNAP1;CD4;NLGN2;PECAM1;NRXN3*
	Focal adhesion	6.73 × 10^−4^	*PPP1CB;PPP1CC;RAP1A;COL4A4;ITGB8;MAPK1;CRK*
	TGF-beta signaling pathway	1.08 × 10^−3^	*SRC;MAPK1;CRK;CD44;PFN2*
	Renal cell carcinoma	1.12 × 10^−3^	*PPP1CB;PPP1CC;FGF7;ITGB8;MAPK1;PIP4K2C;CRK*
	Rap1 signaling pathway	1.32 x 10^−3^	*PPP1CB;PPP1CC;PRKAB2;RAPGEF1;MAPK1*
	ErbB signaling pathway	4.50 × 10^−3^	*ZFYVE16;SP1;MAPK1;INHBA;RGMB*
	VEGF signaling pathway	8.46 × 10^−3^	*PPP1CB;PPP1CC;RAP1A;MAPK1*
	Proteoglycans in cancer	9.37 × 10^−3^	*RAP1A;RAPGEF1;MAPK1;CRK*
**Candidate 5**	**KEGG pathway**	***p*-value**	**Genes**
	Bladder cancer	1.22 × 10^−2^	*VEGFA*
	VEGF signaling pathway	1.75 × 10^−2^	*VEGFA*
	Renal cell carcinoma	2.05 × 10^−2^	*VEGFA*
	Pancreatic cancer	2.22 × 10^−2^	*VEGFA*
	HIF-1 signaling pathway	2.69 × 10^−2^	*VEGFA*
	Focal adhesion	2.69 × 10^−2^	*VEGFA*
	Proteoglycans in cancer	2.96 × 10^−2^	*VEGFA*
**Candidate 6**	**KEGG pathway**	***p*-value**	**Genes**
	Proteoglycans in cancer	2.45 × 10^−3^	*PPP1R12A;ARHGEF12;FZD5;ERBB4;KDR;IGF1R*
	MAPK signaling pathway	3.93 × 10^−3^	*GABRB2;CACNA1C;GABARAP;TRAK2*
	Focal adhesion	1.13 × 10^−2^	*PDGFRA;CACNB4;ERBB4;KDR;CACNA1C;IGF1R*
	Rap1 signaling pathway	5.11 × 10^−2^	*PDGFRA;PPP1R12A;KDR;PARVA;IGF1R*
**Candidate 8**	**KEGG pathway**	***p*-value**	**Genes**
	Hippo signaling pathway	2,00 × 10^−4^	*PPP1CB;LATS2;DLG3;FZD4;TCF7;YWHAZ;TGFBR1;*
	TGF-beta signaling pathway	2.14 × 10^−3^	*CREBBP;TCF7;BAIAP2;TGFBR1;WASF3*
	Breast cancer	5.62 × 10^−3^	*CREBBP;RPS6KB1;NEO1;ACVR2B;TGFBR1*
	Colorectal cancer	7.87 × 10^−3^	*HDAC4;BECN1;RPS6KB1;ADCY2;CALM2;TGFBR1*
	Prostate cancer	9.07 × 10^−3^	*CREBBP;FZD4;TCF7;ADCY2;CALM2*
	Gastric cancer	1.09 × 10^−2^	*RPS6KB1;FZD4;NCOA3;TCF7;PGR;LRP6*
	Wnt signaling pathway	2.19 × 10^−2^	*NRP1;CREBBP;CDC27;ADCY2;TGFBR1;CREB5*

## Supplementary Materials

The following are available online at https://www.mdpi.com/2072-6694/11/12/1924/s1, Figure S1: Novel candidate miRNA validation. Table S1: Sequence and location information for the eight pre-miR candidate. Table S2: List of Custom Assay ID for novel candidate miRNA validation. The sequencing raw data are available online at https://www.ncbi.nlm.nih.gov/sra/PRJNA575520.
