# Supplementary material for: Gathering Novel Circulating Exosomal microRNA in Osteosarcoma Cell Lines and Possible Implications for the Disease
Source: Cancers (Basel). 2019 Dec 3;11(12):1924. doi: 10.3390/cancers11121924 (PMC6966608; doi:10.3390/cancers11121924)
Supplement: Supplementary file 1 [file cancers-11-01924-s001.zip › cancers-631136-suppl-final/Supplementary Figures and Tables.docx]

Communication

Gathering Novel Circulating Exosomal microRNA in Osteosarcoma Cell Lines and Possible Implications for the Disease

Nicola Cuscino ^1,†^, Lavinia Raimondi ^2, †^, Angela De Luca ^2^, Claudia Carcione ^3^, Giovanna Russelli ^1^, Laura Conti ^4^, Jacopo Baldi ^5^, Pier Giulio Conaldi ^1^, Gianluca Giavaresi ^2^ and Alessia Gallo ^1,^*


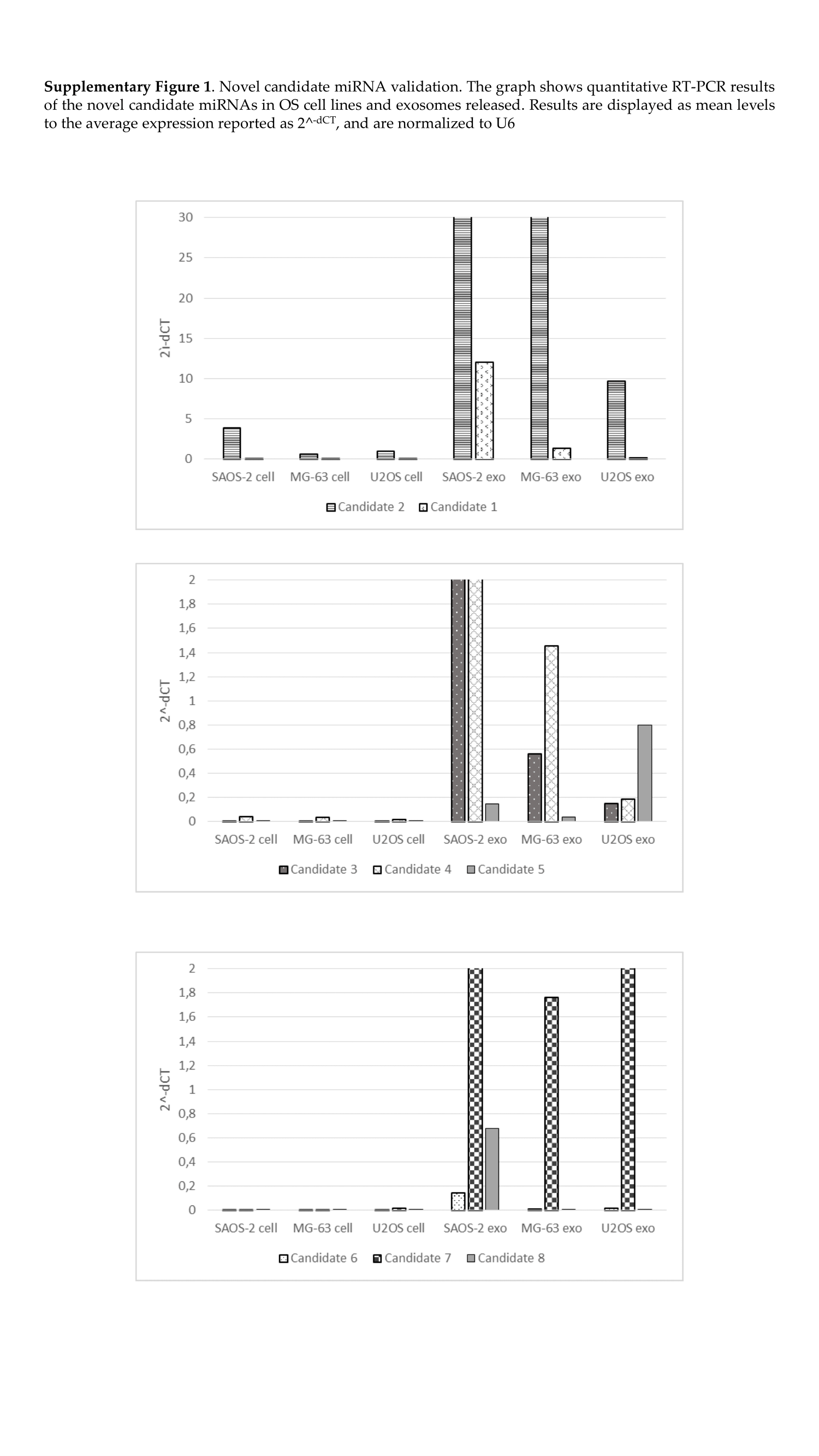


**Supplementary Figure S1. N**ovel candidate miRNA validation. The graph shows quantitative RT-PCR results of the novel candidate miRNAs in OS cell lines and exosomes released. Results are displayed as mean levels to the average expression reported as 2^^−dCT^, and are normalized to U6.

**Supplementary Table S1. Sequence and location information for the eight pre-miR candidate.**

| **novel ID** | **location (hg19)** | **strand** | **Pre-miR sequence** |
| --- | --- | --- | --- |
| pre-miR Candidate 1 | chr9:136204572..136204654 | - | cugaauggcagugggaugucagaacuaauuaaccuuagugucacuauaguuggcauucgacccccacacugcuaaauuugac |
| pre-miR Candidate 2 | chr3:14436198..14436257 | + | uuugggccugguuaguacuuagaugggagaccaccugggaauaacgggugcuguaggcu |
| pre-miR Candidate 3 | chrX:102411053..102411132 | - | ccaaugguaguggguuaucagaacuuauuaacauuaaugucauuaaaguugguauccaccccucacugcuaaauuugac |
| pre-miR Candidate 4 | chr8:56821958..56822010 | - | ccaucugugggauuaugacugaacauguccaagucagaaucccacucaggug |
| pre-miR Candidate 5 | chr8:129022790..129022852 | - | ugcgcaguggcaguaucguagccaaugagguuucaccgaggugcgauuauugcuaauagaaa |
| pre-miR Candidate 6 | chr13:27259470..27259536 | + | agaguagccacuagccacaugucaguucaugcuuuuaaggcuauaugugccuaguggcugcugucu |
| pre-miR Candidate 7 | chr18:59823754..59823805 | - | gaucacuugagcucaggaguuggagaccagucugggcaacaaggugagacc |
| pre-miR Candidate 8 | chr9:89037927..89037972 | - | ucuaucugaggacaauauauuaaauggauuuuuggaaauaggaga |

**Supplementary Table S2.** List of Custom Assay ID for novel candidate miRNA validation.

| **Novel miRNA** | **Assay ID** |
| --- | --- |
| Candidate 1 | CTZTECP |
| Candidate 2 | CT2W7WM |
| Candidate 3 | CT7DPME |
| Candidate 4 | CTDJXPW |
| Candidate 5 | CTEPTAU |
| Candidate 6 | CTH49ZK |
| Candidate 7 | CTNKRRD |
| Candidate 8 | CTPRKCA |
