# Supplementary figures and images for: Gathering Novel Circulating Exosomal microRNA in Osteosarcoma Cell Lines and Possible Implications for the Disease
Source: Cancers (Basel). 2019 Dec 3;11(12):1924. doi: 10.3390/cancers11121924 (PMC6966608; doi:10.3390/cancers11121924)

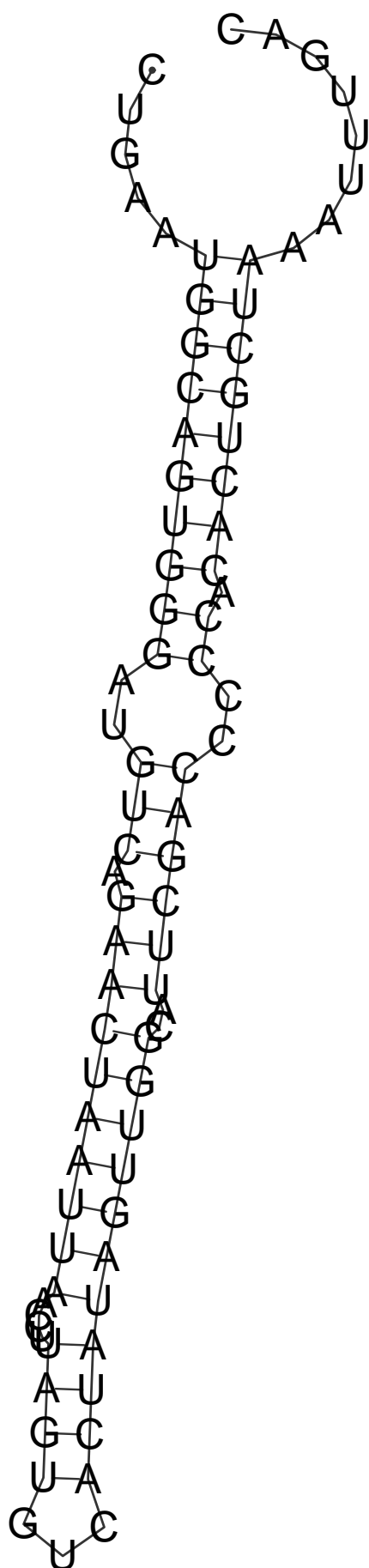

Supplement: Supplementary file 1 [file cancers-11-01924-s001.zip › cancers-631136-suppl-final/Supplementary Material 2-pre_miRNA candidate structures/Candidate 1 pre-miR structure.pdf]

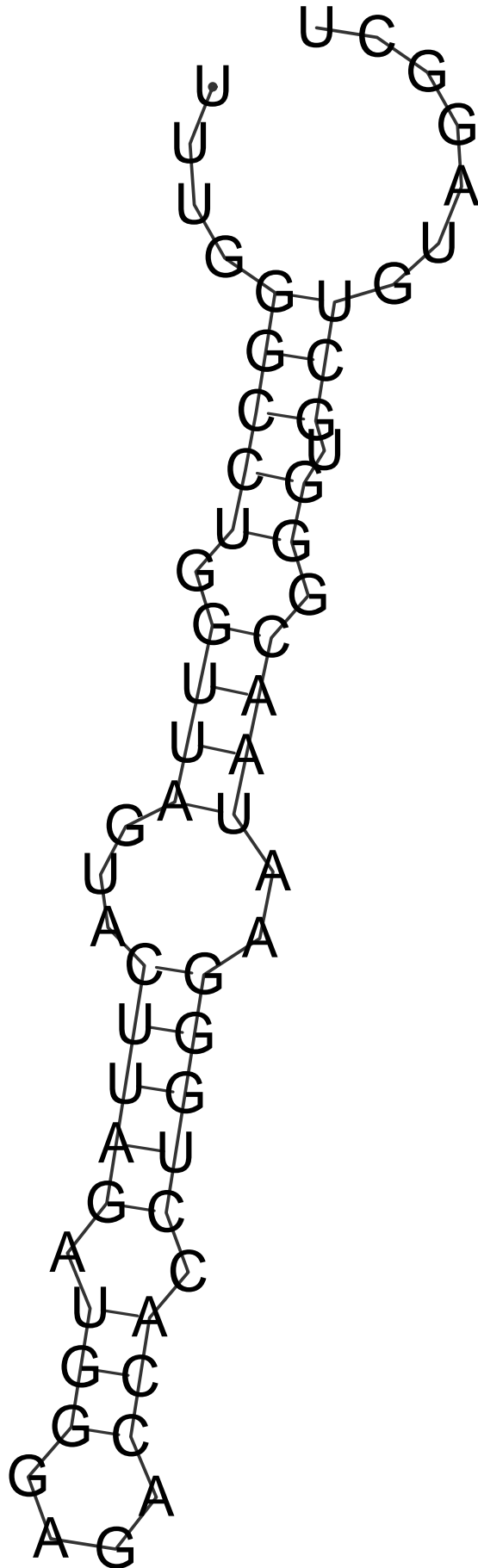

Supplement: Supplementary file 1 [file cancers-11-01924-s001.zip › cancers-631136-suppl-final/Supplementary Material 2-pre_miRNA candidate structures/Candidate 2 pre-miR structure.pdf]

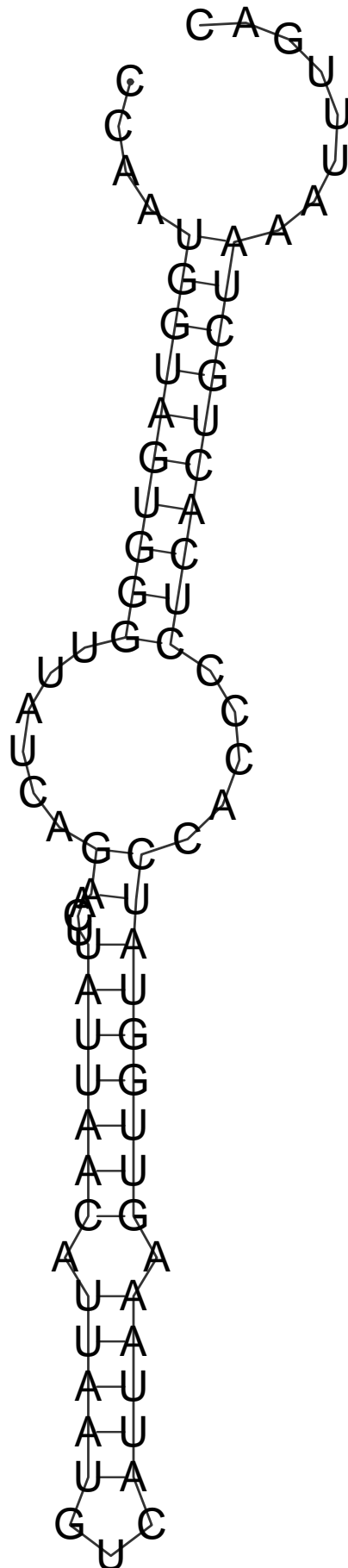

Supplement: Supplementary file 1 [file cancers-11-01924-s001.zip › cancers-631136-suppl-final/Supplementary Material 2-pre_miRNA candidate structures/Candidate 3 pre-miR structure.pdf]

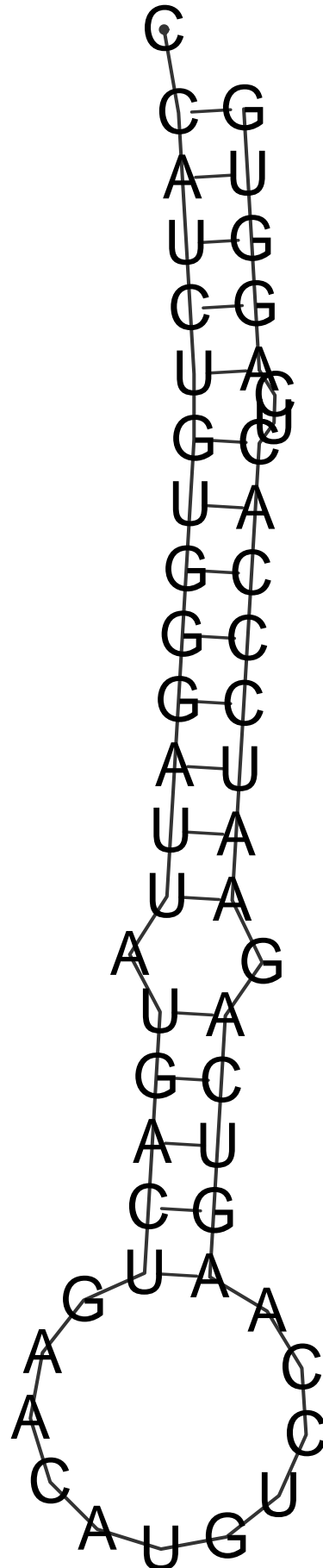

Supplement: Supplementary file 1 [file cancers-11-01924-s001.zip › cancers-631136-suppl-final/Supplementary Material 2-pre_miRNA candidate structures/Candidate 4 pre-miR structure.pdf]

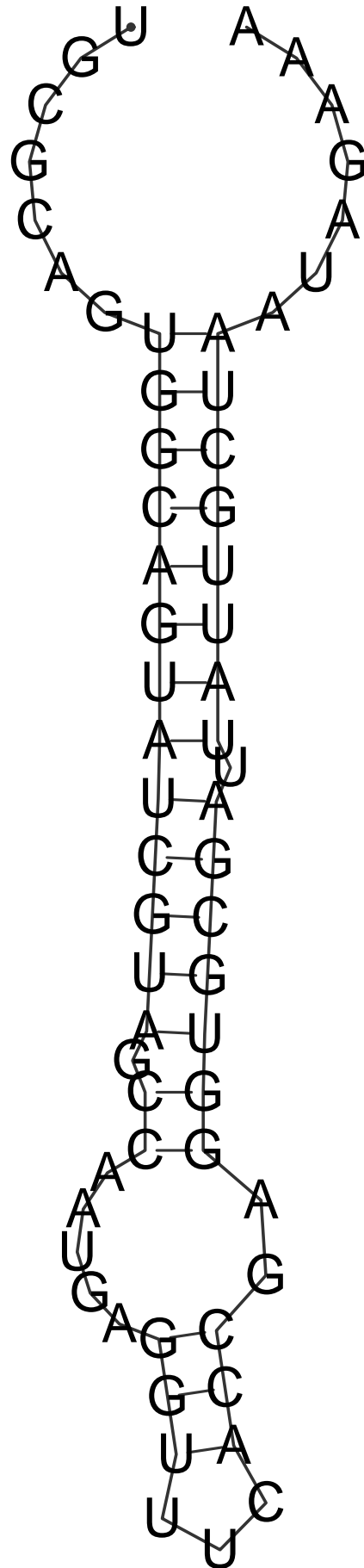

Supplement: Supplementary file 1 [file cancers-11-01924-s001.zip › cancers-631136-suppl-final/Supplementary Material 2-pre_miRNA candidate structures/Candidate 5 pre-miR structure.pdf]

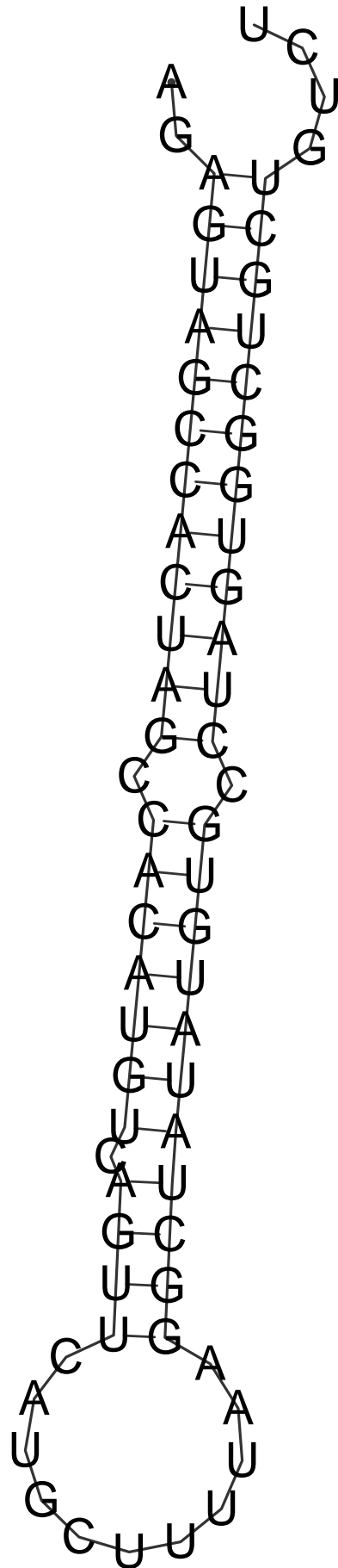

Supplement: Supplementary file 1 [file cancers-11-01924-s001.zip › cancers-631136-suppl-final/Supplementary Material 2-pre_miRNA candidate structures/Candidate 6 pre-miR structure.pdf]

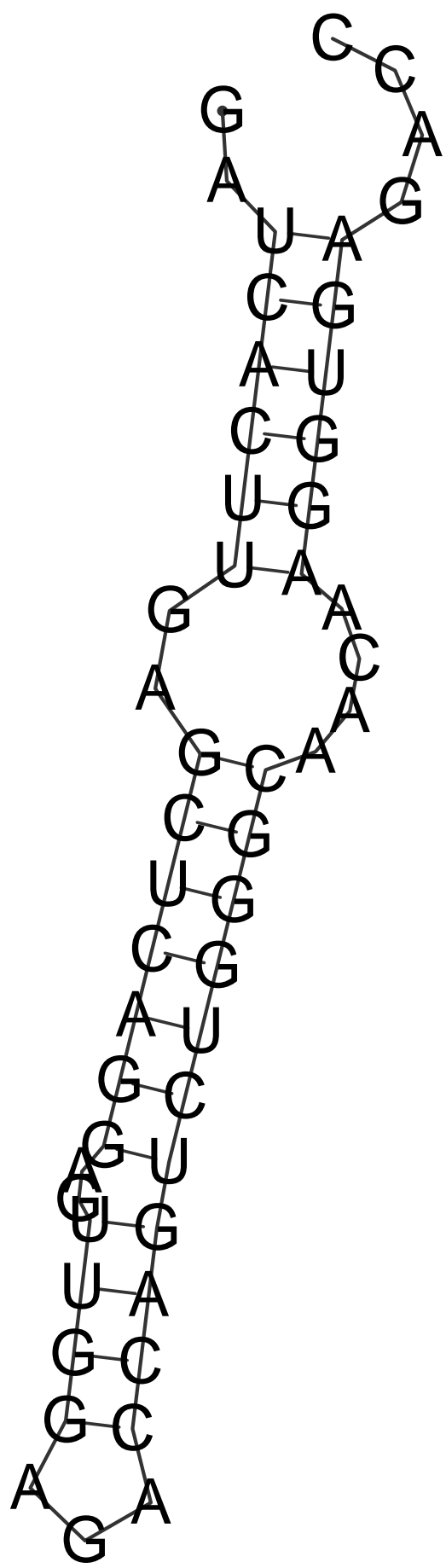

Supplement: Supplementary file 1 [file cancers-11-01924-s001.zip › cancers-631136-suppl-final/Supplementary Material 2-pre_miRNA candidate structures/Candidate 7 pre-miR structure.pdf]

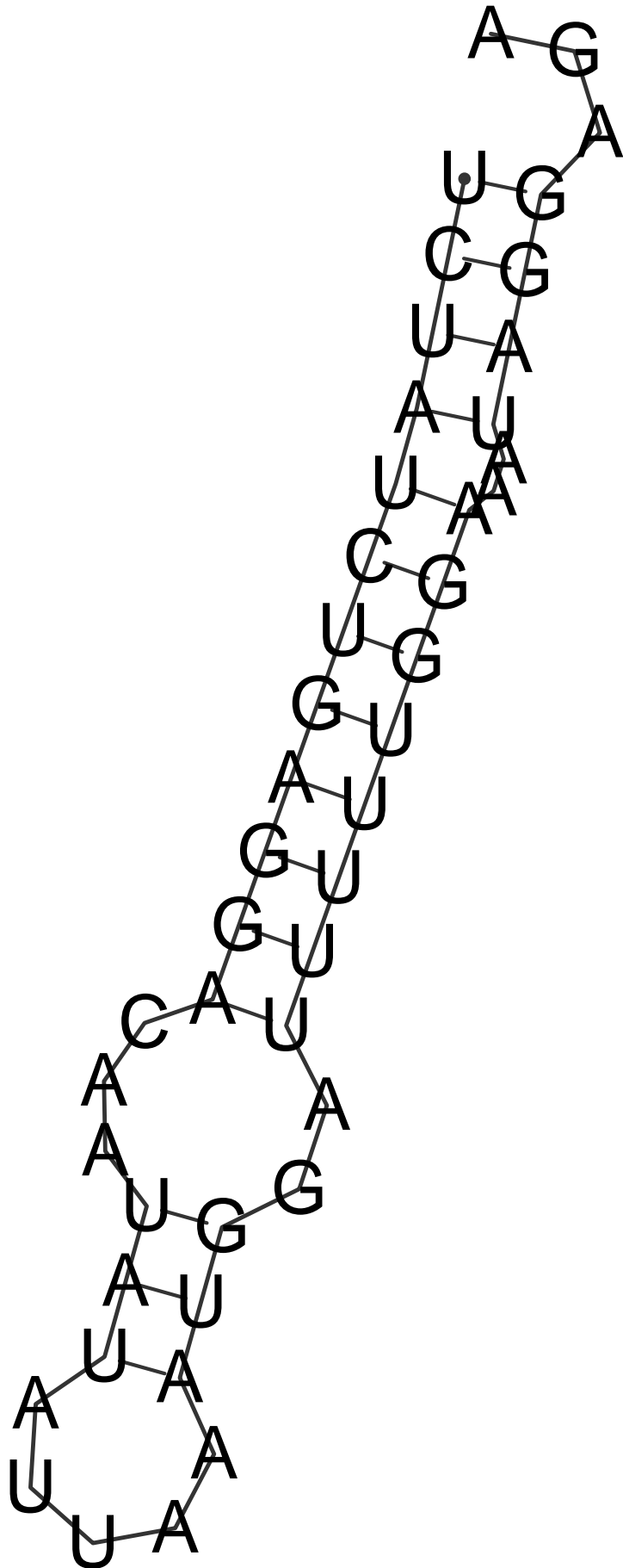

Supplement: Supplementary file 1 [file cancers-11-01924-s001.zip › cancers-631136-suppl-final/Supplementary Material 2-pre_miRNA candidate structures/Candidate 8 pre-miR structure.pdf]
